# Prevalence and determinants of childhood mortality in Nigeria

**DOI:** 10.1186/s12889-017-4420-7

**Published:** 2017-05-22

**Authors:** Sanni Yaya, Michael Ekholuenetale, Godson Tudeme, Shah Vaibhav, Ghose Bishwajit, Bernard Kadio

**Affiliations:** 1 School of International Development and Global Studies, University of Ottawa, Ottawa, ON Canada; 2 Women’s Health and Action Research Centre, Benin City, Nigeria; 3 Hospitals Management Board, Delta State, Asaba, Nigeria; 4 Faculty of Health Sciences, University of Ottawa, Ottawa, ON Canada; 5 Department of Social Medicine and Health Management, Tongji Medical College, Wuhan, China; 6 Faculty of Health Sciences, University of Ottawa, Ottawa, ON Canada

**Keywords:** Zero-inflated negative binomial, Maternal health, Infant mortality, Neonatal mortality, Child mortality, Global health, Nigeria

## Abstract

**Background:**

Childhood mortality has remained a major challenge to public health amongst families in Nigeria and other developing countries. The menace of incessant childhood mortality has been a major concern and this calls for studies to generate new scientific evidence to determine its prevalence and explore predisposing factors associated with it in Nigeria.

**Method:**

Data was obtained from Nigeria DHS, 2013. The study outcome variable was the total number of children lost by male partners and female partners respectively who were married. The difference between the numbers of child births and the number of living children was used to determine the number of children lost. Study variables were obtained for 8658 couples captured in the data set. Descriptive statistics were computed to examine the presence of over-dispersion and zero occurrences. Data were analysed using STATA Software version 12.0. Zero-inflated negative binomial (ZINB) regression analysis was carried out to determine the factors associated with childhood mortality. Results of ZINB were reported in terms of IRR and 95% confidence interval (CI).

**Results:**

The age (mean ± std.) of male and female participants were 36.88 ± 7.37 and 28.59 ± 7.30 respectively. The data showed that 30.8% women reported loss of children and 37.3% men reported the same problem. The study revealed age (years), region, residence, education, wealth index, age at first birth and religion of father and mother as factors associated with childhood mortality. In terms of education, secondary and tertiary educated fathers exhibited 3.8% and 12.1% lower risk of childhood mortality respectively than non-educated fathers. The results showed that the risk of childhood mortality are 26.7%, 39.7 and 45.9% lower among the mothers having primary, secondary and tertiary education respectively than those with no formal education. The mothers living in rural areas experienced 28.3% increase in childhood mortality than those in urban areas, while the fathers in rural areas experienced 33.5% increase in childhood mortality than the urban areas. The risk of childhood mortality was significantly lower in middle, richer and richest (11.1%, 37.5 and 49%) economic quintiles respectively when compared to the risk of childhood mortality with female spouse who are poorest. Similar results were obtained for the fathers, with reduction in the incidence-rate ratio of 3.3%, 20.2 and 28.7% for middle, richer and richest economic quintiles respectively, compared to the poorest status. Furthermore, region and religion were found to be significant factors associated with childhood mortality in Nigeria.

**Conclusion:**

The findings suggested that age, region, residence, education, wealth index, age at first birth and religion of fathers and mothers are key determinants associated with childhood mortality. The correlation between childhood mortality and fathers’ and mothers’ ages were found to increase the incidence of the outcome for every unit increase in age. The converse was however, true for age at first birth which was also statistically significant. The implication of this study is that policy makers and stakeholders in health care should provide for improved living standards to achieve good life expectancy meeting SDG3.

## Background

Childhood mortality is a fundamental measurement of a country’s level of socio-economic and demographic development, and quality of life, especially of families. The Oxford English Dictionary defines a child as “a young human being below the legal age of maturity”. In Nigeria, the legal age of maturity is 18 years [1].

Reports from Nigeria, sub-Saharan Africa and the world at large have revealed that mortality experiences ranging from neonatal mortality, infant and child mortality to maternal mortality are still high [2–4]. Nigeria still has high prevalence of mortalities reflected in infants and children amongst others [5–7].

In Nigeria, the childhood mortality rate stands at 128 per 1000 live births, with large disparities in her different regions [8]. Report from the Nigeria Demographic and Health Survey, 2013 showed that childhood mortality rates range widely across geopolitical zones [9]. Regarding child mortality as a persistent public health challenge in Nigeria and other developing countries, researchers have made immense efforts to identify factors responsible for this menace [10–12].

Studies have shown that mortality rates and risk factors varied by bio-demographic and socio-economic characteristics [13–16]. The factors associated with childhood mortality from studies done in Brazil and America were reported as maternal obesity, maternal malnutrition, maternal short stature and maternal age less than 25 years or greater than 35 years [17, 18]. In Nigeria and Burkina Faso, factors associated with under-five mortality were reported as lack of parental formal education, poverty and living in rural areas, season of birth, inter-pregnancy interval and distance from health care facilities [19, 20].

Tackling the death of children, whether during perinatal, early or late neonatal, childhood or adolescent age is posing a difficult task in Nigeria. Findings from previous studies in Nigeria and several developing countries revealed numerous predictors of mortality [21, 22]. These findings triggered intervention initiatives which aimed to identify the factors responsible for the high mortality rates and the most appropriate techniques for tackling them. Nigeria is yet to meet the Sustainable Development Goals (SDGs) targets, regardless of national and international implementation projects on the reduction of mortality.

A key factor such as inadequate health care services remains a frontline problem in Nigeria. SDGs identify the minimum requirements to improve the general wellbeing of a population [23–25]. One of the stated goals is the Good Health and Well-being (SDG3). Ensuring healthy lives and promoting the well-being of the general population at all ages is essential to sustainable development.

Progress has been made on increasing access to basic needs to enhance life expectancy, but more effort is needed to fully eradicate a wide range of barriers and address a plethora of persistent and emerging health issues. Statistics show that most sub-Saharan countries in Africa, including Nigeria, have a lot to do in achieving the SDG3. More research is therefore needed to inform the formulation of policies and implementation of programs for appropriate health intervention. There is need for data on the lifetime experience of childhood mortality. Thus, this study examined the factors associated with childhood mortality in Nigeria using NDHS, 2013 dataset.

## Methods

### Data source

Data was obtained from Nigeria DHS, 2013. The survey was done across the entire population. The country was divided into the six geopolitical zones which in turn are made up of the 36 states and the federal capital territory. From the 2006 Population census implementation in Nigeria, each region was subdivided into Enumeration Areas. The sample frame was drawn from a list of the enumeration areas. The sampling method for the 2013 Nigeria DHS was a three-stage stratified random sample. In the first stage, each State was stratified into rural and urban areas, and this brought a list of localities. In the second stage, one enumeration area was selected at random from a selected list of localities and the resulting list of households gave the list for the selection of households at the last stage. In the third stage, forty-five households were chosen in every rural and urban cluster through systematic sampling using the sample frame.

### Data extraction

Our study focused on the total number of children lost by male partners and female partners respectively who were married. The difference between the numbers of child births and the number of living children was used to determine the number of children lost. Study variables were obtained for 8658 couples captured in the data set.

### Ethical clearance

The demographic and health survey program has its own standards for protecting the privacy of participants. Prior to each interview, the interviewer is made to read the consent statement and inform the participant of voluntary participation and that he/she has the freewill to terminate the interview at any stage of the process. Furthermore, the ICF International certifies that the survey complies with the United States Department of Health and Human Services rules for the protection of participants and ensures that the survey follows the laws and regulations of the nation. However, approval for this study was not needed since the data is secondary.

### Data analysis

Data analysis was conducted using STATA Software version 12.0. Zero-inflated negative binomial regression (ZINB) analysis was carried out to determine the risk factors of childhood mortality. Descriptive statistics were computed to examine the presence of over-dispersion and zero occurrences. Results of ZINB were reported in terms of IRR and 95% confidence interval (CI). *p*-value of <0.05 was considered statistically significant. Zero-inflated negative binomial regression is for modeling count variables with excess zeros and it is used for over-dispersed count outcome variables. Furthermore, theory suggests that a separate process from the count values generates the excess zeros and that the excess zeros can be modeled independently.

## Results

### Descriptive statistics

The age (mean ± std.) of male and female participants were 36.88 ± 7.37 and 28.59 ± 7.30 respectively, showing that male partners had higher mean age than their female counterparts. The data showed that 2667 (30.8%) women reported loss of children compared to 3231 (37.3%) men. The test of proportionality showed statistical significant difference between men and women who had lost children (*z* = −9.04; 95% CI: -0.079, −0.051; *p* < 0.001).

Table 1 showed the frequency distribution of the study variables. About one-third of female partners were in their late 20’s and only about one-tenth of them were above 39 years old. But the age interval reported by male partners showed that the men were predominantly above 30 years old. Couples from South East were least captured in the study while North West had the highest representation of about one-third. Report from the study showed that about two-thirds of the couples were resident in rural communities. In addition, more women than men were reported as having no formal educational qualifications. Also, two times the male partners (14.6%) had higher educational qualifications compared to the female partners (7.5%); this clearly shows that male partners were more educated than their female counterparts. Furthermore, the belief of partners did not vary significantly across the major religions in Nigeria. The report also showed that a uniform spread (about one-fifth) was obtained across the wealth index level of couples. Current unemployed male partners were minimal (4.2%) when compared to the female partners (32.6%). More so, about 95.8% of male partners were currently employed while only about two-thirds of female partners were currently employed. This shows that more economic strength rests on the male partners than the female partners.

**Table 1:** Study variables of participants

Variable	Frequency	Percentage
Age group of female partners		
15–19	872	10.1
20–24	1674	19.3
25–29	2305	26.6
30–34	1703	19.7
35–39	1318	15.2
40–44	608	7.0
45–49	178	2.1
Total	8658	100.0
Age group of male partners		
15–19	20	0.2
20–24	323	3.7
25–29	1113	12.9
30–34	1636	18.9
35–39	1945	22.5
40–44	1785	20.6
45–49	1836	21.2
Total	8658	100.0
Region of couples		
North Central	1412	16.3
North East	1719	19.9
North West	2851	32.9
South East	515	5.9
South South	1043	12.0
South West	1118	12.9
Total	8658	100.0
Residence of couples		
Urban	2821	32.6
Rural	5837	67.4
Total	8658	100.0
Education of female partners		
No formal education	3942	45.5
Primary	1767	20.4
Secondary	2301	26.6
Higher	648	7.5
Total	8658	100.0
Education of male partners		
No formal education	2757	31.8
Primary	1947	22.5
Secondary	2688	31.0
Higher	1266	14.6
Total	8658	100.0
Religion of female partners		
Christianity	3421	39.8
Islam	5099	59.3
Traditional	80	0.9
Total	8600	100.0
Religion of male partners		
Christianity	3366	39.1
Islam	5132	59.6
Traditional	108	1.3
Total	8606	100.0
Wealth Index of couples		
Poorest	1963	22.7
Poorer	1891	21.8
Middle	1576	18.2
Richer	1613	18.6
Richest	1615	18.7
Total	8658	100.0
Current working status of female partners		
Employed	5807	67.4
Not employed	2803	32.6
Total	8610	100.0
Current working status of male partners		
Employed	8276	95.8
Not employed	367	4.2
Total	8643	100.0

### Assessing the effect of predictor variables for female partners in childhood mortality using the ZINB model

Table 2 showed that age (years) of female partners was statistically significant with childhood mortality. For every unit increase in age, there was 8.1% increase in the incidence of childhood mortality (IRR = 1.081; 95%CI = 1.073–1.089; *p* < 0.001). For the geographical zones/regions in the ZINB model, the risk of mortality among children increased by 21.5% in North East (IRR = 1.215; 95% CI: 1.012–1.460; *p* < 0.001), 38.3% in North West (IRR = 1.383; 95% CI: 1.161–1.647; *p* < 0.001), 76% in South East (IRR = 1.760; 95% CI: 1.405–2.205; *p* < 0.001), 34.1% in South South (IRR = 1.341; 95% CI: 1.086–1.657; *p* < 0.001), and 36.4% in South West (IRR = 1.364; 95% CI: 1.117–1.667; *p* < 0.001) compared to the risk of mortality among children in the North Central. For location of residence, there was a 28.3% increase in the risk of mortality among children of female partners in rural locations (IRR = 1.283; 95% CI: 1.130–1.458; *p* < 0.001) compared to the risk of mortality among children of female partners in urban locations.

**Table 2:** Parameter estimates in the Zero-inflated negative binomial regression model for childhood mortality among female partners

Variable	IRR	Std.err	95% CI	P
Age (years)	1.081	0.004	1.073–1.089	<0.001***
Region				
North Central (ref)	1			
North East	1.215	0.114	1.012–1.460	0.037***
North West	1.383	0.123	1.161–1.647	<0.001***
South East	1.760	0.203	1.405–2.205	<0.001***
South South	1.341	0.145	1.086–1.657	0.007***
South West	1.364	0.140	1.117–1.667	0.002***
Residence				
Urban (ref)	1			
Rural	1.283	0.083	1.130–1.458	<0.001***
Education				
No Education (ref)	1			
Primary	0.733	0.046	0.648–0.830	<0.001***
Secondary	0.603	0.048	0.515–0.705	<0.001***
Tertiary	0.541	0.074	0.413–0.707	<0.001***
Wealth Index				
Poorest (ref)	1			
Poorer	0.973	0.053	0.875–1.083	0.619
Middle	0.889	0.071	0.760–1.040	0.141
Richer	0.625	0.054	0.527–0.742	<0.001***
Richest	0.510	0.059	0.406–0.641	<0.001***
Age at 1st birth (years)	0.926	0.006	0.914–0.938	<0.001***
Religion				
Christianity (ref)	1			
Islam	1.184	0.108	0.990–1.416	0.064
Traditionalist	1.432	0.142	1.179–1.740	<0.001***
Employment				
No (ref)	1			
Yes	1.096	0.054	0.996–1.207	0.061

***Significant at p < 0.05; IRR = Incidence-rate ratios. Vuog test of ZINB vs standardized negative binomial *z* = 4.97; *p* < 0.001. McFadden Pseudo R^2^ = 0.322

For the educational level categories of female partners in the ZINB model, the risk of mortality among children of female partners reduced by 26.7% in Primary (IRR = 0.733; 95% CI: 0.648–0.830; *p* < 0.001), 39.7% in Secondary (IRR = 0.602; 95% CI: 0.515–0.705; *p* < 0.001), and 45.9% in Tertiary (IRR = 0.541; 95% CI: 0.413–0.707; *p* < 0.001) compared to female partners with no formal education. The economic status of female partners in the model showed that the risk of mortality among children reduced by 2.7% in Poorer (IRR = 0.973; 95% CI: 0.875–1.083; *p* = 0.619), 11.1% in Middle (IRR = 0.889; 95% CI: 0.760–1.040; *p* = 0.141), 37.5% in Richer (IRR = 0.625; 95% CI: 0.527–0.742; *p* < 0.001) and 49% in Richest (IRR = 0.510; 95% CI: 0.406–0.641; *p* < 0.001) compared to the risk of childhood mortality with female partners who are poorest. For every unit increase in the age (years) of female partners at 1st birth, there was 7.4% reduction in the incidence of childhood mortality (IRR = 0.926; 95%CI = 0.914–0.938; *p* < 0.001). Religion of female partners impacted mortality among children; childhood mortality increased by 18.4% in Islam (IRR = 1.184; 95% CI: 0.990–1.416; *p* < 0.064) and 43.2% in Traditionalist (IRR = 1.432; 95% CI: 1.179–1.740; *p* < 0.001) compared to Christianity. Children of employed women had 9.6% increase in the risk of childhood mortality (IRR = 1.096; 95% CI: 0.996–1.207; *p* = 0.061) compared to unemployed female partners. The proportion of the variance of the outcome variable that is explained by the factors was (McFadden) Pseudo R^2^ = 0.322, which showed that the fitness of the model was satisfactory.

### Assessing the effect of predictor variables for male partners in childhood mortality using the ZINB model

From Table 3, for every unit increase in age, there was 8.6% increase in childhood mortality (IRR = 1.086; 95%CI = 1.078–1.093; *p* < 0.001). For the geographical region of male partners in the ZINB model, the risk of mortality among children increased by 12.5% in North East (IRR = 1.125; 95% CI: 0.958–1.322; *p* = 0.152), 35.8% in North West (IRR = 1.358; 95% CI: 1.156–1.597; *p* < 0.001) and 24% in South South (IRR = 1.240; 95% CI: 0.993–1.548; *p* = 0.057) when compared to North Central. However, there was a 13% reduction in South East (IRR = 0.870; 95% CI: 0.651–1.160; *p* = 0.342) and a 24.9% reduction in South West (IRR = 0.751; 95% CI: 0.585–0.964; *p* = 0.024) when compared to North Central. For location of residence of male partners, there was a 33.5% increase in the risk of mortality among children in rural locations (IRR = 1.335; 95% CI: 1.174–1.518; *p* < 0.001) compared to the risk of mortality among children in urban locations. For the educational level of male partners in the ZINB model, the risk of mortality among children reduced by 3.8% in Secondary (IRR = 0.962; 95% CI: 0.855–1.083; *p* = 0.520) and 12.1% in Tertiary (IRR = 0.879; 95% CI: 0.747–1.034; *p* = 0.119) levels compared to no formal education.

**Table 3:** Parameter estimates in the Zero-inflated negative binomial regression model for childhood mortality among male partners

Variable	IRR	Std.err	95% CI	P
Age (years)	1.086	0.004	1.078–1.093	<0.001***
Region				
North Central (ref)	1			
North East	1.125	0.092	0.958–1.322	0.152
North West	1.358	0.112	1.156–1.597	<0.001***
South East	0.870	0.128	0.651–1.160	0.342
South South	1.240	0.140	0.993–1.548	0.057
South West	0.751	0.096	0.585–0.964	0.024***
Residence				
Urban (ref)	1			
Rural	1.335	0.087	1.174–1.518	<0.001***
Education				
No Education (ref)	1			
Primary	1.074	0.051	0.979–1.179	0.131
Secondary	0.962	0.058	0.855–1.083	0.520
Tertiary	0.879	0.073	0.747–1.034	0.119
Wealth Index				
Poorest (ref)	1			
Poorer	1.016	0.048	0.926–1.114	0.738
Middle	0.967	0.062	0.853–1.096	0.601
Richer	0.798	0.073	0.667–0.954	0.013***
Richest	0.713	0.090	0.558–0.913	0.007***
Age at 1st birth (years)	0.973	0.003	0.968–0.979	<0.001***
Religion				
Christianity (ref)	1			
Islam	1.059	0.115	0.856–1.311	0.596
Traditionalist	1.531	0.169	1.232–1.901	<0.001***
Employment				
No (ref)	1			
Yes	0.997	0.072	0.865–1.150	0.972

***Significant at p < 0.05; IRR = Incidence-rate ratios. Vuog test of ZINB vs standardized negative binomial *z* = 7.17; *p* < 0.001. McFadden Pseudo R^2^ = 0.309

On the contrary, there was a 7.4% increase in the risk of mortality among children of male partners in Primary (IRR = 1.074; 95% CI: 0.979–1.179; *p* = 0.131) compared to male partners with no formal education. For the economic status category, the model showed that the risk of mortality among children of male partners was reduced by 3.3% in Middle (IRR = 0.967; 95% CI: .0853–1.096; *p* = 0.601), 20.2% in Richer (IRR = 0.798; 95% CI: 0.667–0.954; *p* = 0.013) and 28.7% in Richest (IRR = 0.713; 95% CI: 0.558–0.913; *p* = 0.007) compared to the poorest. But result showed a 1.6% increase in Poorer (IRR = 1.016; 95% CI: 0.926–1.114; *p* = 0.738) compared to the Poorest which was not statistically significant. For every unit increase in the age (years) at 1st birth, there was 2.7% reduction in childhood mortality (IRR = 0.973; 95% CI = 0.968–0.979; *p* < 0.001). Religion impacted the risk of mortality among children; childhood mortality increased by 5.9% in Islam (IRR = 1.059; 95% CI: 0.856–1.311; *p* = 0.596) and 53.1% in Traditionalist (IRR = 1.531; 95% CI: 1.232–1.901; *p* < 0.001) compared to Christianity. Children of employed male partners had 0.3% reduction in mortality rates (IRR = 0.997; 95% CI: 0.865–1.150; *p* = 972) compared to unemployed male partners. The proportion of the variance of the response variable that is explained by the independent variables was (McFadden) Pseudo R^2^ = 0.309, which showed that the fitness of the model was satisfactory.

## Discussion

This study has shown the prevalence and factors associated with childhood mortality in Nigeria. The study reported that for every unit increase in the ages of male and female partners, the risk of childhood mortality increases and this is similar to the findings of Adetoro & Amoo [26]. From the results, couples who had their first child at an earlier age were more susceptible to the occurrence of childhood mortality. These findings show consistency with the results obtained by Nazrul, Kamal & Korban [27]. Findings from the study reveal that couples with formal education experienced lower childhood mortality than those without formal education. For example, fathers with secondary and tertiary education had 3.8% and 12.1% reduction in childhood mortality respectively than non-educated fathers. It has been consistently claimed that mother’s education is a prominent factor in explaining risk of childhood mortality.

The results showed that the risk of childhood mortality are 26.7%, 39.7% and 45.9% lower among the mothers having primary, secondary and tertiary education respectively than those who have no formal education. Studies assert that mothers’ education helps in teaching quality health practices and improving health behaviour such as feeding habits and child care. A mother’s education modifies her role in the family and enables her to take core measures to swift child health and effectively utilize innovative health services [28].

The incidence-rate ratio for the place of residence reveals that the chance of childhood mortality is lower in the urban area than in rural area. The mothers living in rural areas experienced 28.3% increase in childhood mortality than the urban areas, while the fathers living in rural areas experienced 33.5% increase than the urban areas [29, 30]. It is perceived that urban areas are connected not only with quality health care services, but also with good education and employment opportunities for mothers, implying a lower experience in childhood death. Childhood mortality was significantly lower in middle, richer and richest (11.1%, 37.5% and 49%) economic status respectively when compared to the risk of childhood mortality with female spouse who are poorest. Similar results were obtained for the fathers with reduction in the incidence-rate ratio (3.3%, 20.2% and 28.7%) for middle, richer and richest economic status respectively compared to the poorest status [30, 31]. Furthermore, region and religion were found to be significant factors in the risk of childhood mortality in Nigeria and this is consistent with the study of Antai [32, 33].

### Strengths and limitations

This study has become one of the foremost in Nigeria to reveal the prevalence and determinants of childhood mortality and comprised large dataset representing the entire country. In addition, the non-response rate was very low, less than 10%. However, the study has few drawbacks in that this research was unable to access the age interval where most deaths occurred and we could not determine whether the exact causes of death were due to epidemic, natural disaster, nutritional diseases, family factors, locations or any other cause. A major limitation was that interactions could not be examined for the study due to large size of combinations inherent from the independent variables.

## Conclusion

Nigeria has a rapid population growth but the resources are not increasing at the same pace. As a result, the major portion of the population is faced with low chances of survival. The poverty inherent in the rapid population growth has led to lack of formal education, child labour and sometimes other serious health problems that can increase mortality rate. This study applied the nationally representative data from the Nigeria Demographic and Health Survey, 2013 to explore factors associated with childhood mortality in Nigeria. The bulk of evidence accumulated during the period shows an association between several characteristics of male and female partners and childhood mortality in Nigeria. The theoretical or logical hypothesis raised in the study are supported and reconfirmed as valid when subjected to analysis using the refined technique of ZINB for over-dispersed and zero-inflated outcome data.

The findings suggest that age, region, residence, education, wealth index, age at first birth and religion of fathers and mothers are prominent factors associated with childhood mortality. The association between childhood mortality and fathers’ and mothers’ ages was found to increase the incidence of the outcome for every unit increase in age. The converse was however true for age at first birth which was also statistically significant. Childhood mortality was found to be higher for rural dwellers.

Strategies to reduce the risk of childhood mortality in the country should involve more investments on parents’ empowerment programs in terms of education and economic opportunities, which could reduce poor health outcomes of their children. The implication of this study is that policy makers and stakeholders in health care will be exceedingly optimistic about the ability of health campaigns to solely encourage utilization of appropriate living standards to improve life expectancy.
